# Marital status and suicide risk: Temporal effect of marital breakdown and contextual difference by socioeconomic status

**DOI:** 10.1016/j.ssmph.2021.100853

**Published:** 2021-06-20

**Authors:** Erik Oftedahl Næss, Lars Mehlum, Ping Qin

**Affiliations:** National Center for Suicide Research and Prevention, Institute of Clinical Medicine, Faculty of Medicine, University of Oslo, Sognsvannsveien 21, N-0372, Oslo, Norway

**Keywords:** Suicide, Marital status, Marriage, Marital separation, Registry data, Socioeconomic status

## Abstract

Research has shown that people who have never been married, divorced, or widowed are at an increased risk of suicide compared with those who are married, but we have little knowledge as to how this elevated risk is modified by socioeconomic factors, and little research has studied the risk among persons enduring a marital separation. This study addressed these issues with individual-level data from Norwegian national registers. All suicide cases in people above 18 years that took place in the period 1992–2012 (n = 11 051) were compared with living controls (185 685) matched on sex and age via a nested case control design, and suicide risk associated with marital status was assessed with conditional logistic regression. The results showed that, compared with a status of being married, suicide risk was highly associated with a status of being never married, separated, divorced, or widowed, even after adjustment for income-level, educational attainment, centrality of residence, and immigration status. The strongest effect was seen for a separated status; compared to the married, separated persons were fully 6.06 times more apt to die by suicide, and the effect was strongest in the 30 days following a separation. The observed significant associations remained but differed in strength by sex and age, and there were significant deviations by personal socioeconomic status. Most notably, the increased risk was higher for never-married persons with low educational attainment or income. However, most interaction effects (10/16) did not yield significant results. In conclusion, suicide risk is strongly associated with a single status of any form with the highest risk during a marital separation, but the increased risk varies in strength according to individual-level factors. The stress and loss of support induced by marital dissolution are important contributing risk factors for suicide, and persons with low income may be especially vulnerable.

## Highlights

• Single status of any form was associated with increased risk of suicide.
• The risk was highest during the first month of a marital separation.
• A separated status had the strongest effect in middle-aged adults of both sexes.
• The risk for individuals who had never married differed by income and education.

## 1 Introduction

Social bonds and attachments play critical roles in human functioning; people thrive under strong long-lasting relationships and feel distressed when these are threatened or broken (Baumeister & Leary, 1995). Marital unions are arguably the strongest bonds formed by non-kin and are associated with reduced risks of physical and psychiatric disorders (Kiecolt-Glaser & Newton, 2001; Williams et al., 1992), higher well-being, and increased financial satisfaction (Stack & Eshleman, 1998).

Some of the mentioned differences between the married and single population may be accounted for by matrimonial selection and selection into divorce (Idstad et al., 2015; Mastekaasa, 1992), but components of marriage might play important causal roles. Being married may increase the likelihood of having a confidant (Lowenthal & Haven, 1968) and reduce the risk of loneliness (Stack, 1998). Positive social control and care from a partner may lead to a healthier lifestyle (Umberson, 1992), and a shared economy facilitates the accumulation of wealth (Waite & Gallagher, 2001). Married persons usually experience higher levels of social support (Ross, 1995; Soulsby & Bennett, 2015), which is believed to enhance general well-being in daily life through positive affect and recognition of self-worth, and to buffer adverse psychological and physiological reactions that may arise from stressful live-events and conditions (Cohen & Wills, 1985). Directly related to suicide, Durkheim's theory of social integration proposes that social ties reduce the likelihood of egoistical suicide by subverting individualistic tendencies through identity, shared values, and obligations (Durkheim, 1897). Furthermore, according to Joiners interpersonal theory of suicide, thwarted belonging is one of the necessary psychological factors seen on the pathway to suicide (Van Orden et al., 2010).

Over the last decades, a large body of research has shown a strong association between a status of being never married, divorced, or widowed, and the risk of suicide. This risk is highest for a divorced status and seems to be more pronounced for men, especially for a widowed status (Kyung-Sook et al., 2018). Moreover, some studies have indicated that the increased risk is strongest in early adulthood to mid-life (Mastekaasa, 1995; Wyder et al., 2009; Yip, 1998; Yip et al., 2012).

Marital breakdown not only implies the loss of the benefits of marriage, it is also frequently associated with conflict, self-incrimination, economic problems, and reduced contact with children (Amato, 2000). The first period after a separation might be a period of shock, undecidedness, and uncertainty about the future, which could add to the burden. It has been hypothesized that marital dissolution may lead to a state of crisis characterized by increased levels of psychological stress that is usually alleviated with time (Booth & Amato, 1991). As persons enduring a marital separation usually are closer to the actual relationship breakdown than people in a divorced status, their risk of suicide may, therefore, possibly be higher than for divorced people. To our awareness, however, few studies have addressed the risk of suicide for people in a separated status. Studies from Australia have suggested a higher risk for a separated than a divorced status, especially for men (Cantor & Slater, 1995; Wyder et al., 2009). However, both a Danish register study and a US study found the risk among the separated to be approximately the same as for the divorced (Agerbo, 2005; Kposowa et al., 2020), while another US study found no increased risk associated with being separated as compared to being married (Cook, 2019). There has also been little research conducted on the temporal effect of marital breakdown. A large psychological autopsy study in the US found a more pronouncedly increased risk among suicide decedents being divorced within the last 2–3 years than those divorced for a longer time (Stack & Scourfield, 2015). An Australian study found the risk of suicidal ideation and suicide plans or attempt to be most elevated the first two years after a separation (Batterham et al., 2014). However, to our awareness, no study has investigated the temporal effect of separation on suicide in detail.

Socioeconomic determinants are positively associated with material, personal and intrapersonal resources that may facilitate coping and problem solving (Côté et al., 2010; Liebler & Sandefur, 2002; Lowenthal & Haven, 1968; Stansfeld et al., 1997), which could imply that persons with low educational attainment or income are less equipped to handle stressors, such as marital dissolution and bereavement, and that they may gain a stronger compensatory effect from social support and other resources attained through marriage. On the other hand, it has been argued that people with high educational attainment could gain stronger suicide protection from marriage, as people tend to marry within their own social stratum and the combined resources may interact positively (Denney, 2014). Studies that have investigated how marriage and educational attainment interact to influence suicide risk have shown contradictory results (Denney, 2014; Lorant et al., 2005), and to our knowledge, no studies have analyzed the interaction between level of education or income for each of the single marital statuses separately.

This study aims to fill the above mentioned knowledge gaps by utilizing individual data interlinked from several Norwegian population registers for persons who died from suicide during 1992–2012 and comparison subjects that were matched to the cases on sex and age via a nested case-control design. Our objectives are to investigate how suicide risk is related to marital status in the Norwegian population, and to examine how sex, age, income-level, and educational attainment could modify the associations.

## 2 Methods

### 2.1 Data sources

The study was based on the entire population of Norway. Individual data from three Norwegian longitudinal registers were interlinked through the unique personal identification number possessed by everyone registered in the Central Population Register (CPR) (National Population Register). CPR is administered by the Norwegian Tax administration and contains information on all persons who reside or have resided in Norway, including date and place of birth, status of residence, and citizenship. The statistics Norway Event Database (FD-Trygd) contains running data from 1992 and onwards on factors related to marital status, address of residence, demographics, labor, and social benefits (SSB, 2014). The Cause of Death Register is administered by the Institute of Public Health and contains data on the date and cause of death of persons who dies in Norway and Norwegian citizens who dies abroad (NIPH, 2019). The causes of death are classified according to the WHO's International Classification of Diseases-system 9th revision (ICD-9, suicide coded E959-E959) from 1985, and the 10th revision (ICD-10, suicides code X60-X84, Y870) from 1996.

### 2.2 Study subjects and design

All registered suicide cases in Norway in the period 1.1.1992–31.12.2012 were identified, and matched on sex and birth date, through a nested case-control design, with up to 20 live controls for each suicide case (Clayton et al., 1993). Comparison subjects were selected from a representative 25% sample of the Norwegian population by incidence density sampling (i.e. from the population at risk for suicide at the suicide case date) (Clayton et al., 1993), to minimize possible bias induced by variables that may change over time during the study period. If there were more than 20 eligible controls, 20 were selected randomly, otherwise, all were included. Subjects below 18 years were excluded, as very few are married at that age, and so were persons who resided outside of Norway at the case date, as they were not active residents of Norway and thus had a high proportion of missing socioeconomic information. That left 11 051 cases and 185 685 controls included as study subjects.

### 2.3 Variables of interest

#### 2.3.1 Marital status

Marital status was the main variable of interest and was categorized to reflect status at case date: a) married, b) never married, c) separated, d) divorced, e) widowed, and f) unknown. The categories are mutually exclusive, so for instance, a person who was previously separated, divorced, or widowed, but was currently married at the time of suicide or matching was only in the married category. A separated status was further divided by days elapsed since the separation was filed: 0–30, 31–92, 93–183, 184–365, and >365. According to Norwegian jurisdiction, a condition to file for divorce under normal circumstances is to have filed for separation and subsequently lived apart for minimum one year (Separasjon og skilsmisse, 2019). Being married was the reference category in all analyses.

### 2.3.2 Other variables

Covariates under consideration included educational attainment, income-level, place of residence, and immigration background. Information on highest education attained by the last 1st of October was extracted from the FD-Trygd database and divided into three categories: a) secondary school, b) high school, and c) bachelor degree and higher. In Norway, secondary school is mandatory and lasts for 10 years (9 years for persons born before 31.12.1990), while high school is either a 3-year lap as a preparation for higher education, or a 2+2-year lap of theory of practice to achieve a certificate of apprenticeship. Information on taxable gross income in the year preceding case date was extracted from the FD-Trygd database. To adjust for inflation, income was divided by a standardized amount, the “G”, which is a standard sum of money used as a basis for calculation of pensions and social security benefits in Norway. The G is adjusted annually according to the expected growth of salaries (NAV, 2019), with 1 G being 37 300 NOK in 1993 and 79 216 NOK in 2011. Income was then categorized into Low (< 3G), Medium (3 - 6G), and High (> 6G) income. Information on place of residence was extracted from the FD-Trygd register, and according to the classification of centrality of municipalities made by the Statistics Norway (SSB, 2008), four categories were made, from least central to most central, based on the population size of the nearest urban area and travel time to this area. Immigration background status was extracted from CPR and contains six categories: a) born in Norway with two Norwegian-born parents, b) foreign-born with two foreign-born parents, c) born in Norway with two foreign-born parents d) foreign-born with one Norwegian-born parent, e) Norwegian-born with one foreign-born parent, and f) foreign-born with two Norwegian-born parents.

### 2.4 Statistical analyses

All analyses were conducted with the statistical software package StataSE 16. Conditional logistic regression was performed with the clogit function to yield odds ratios (ORs) with 95% confidence intervals (95%CI), and the Wald-test to examine if the ORs were significantly different from the reference category. ORs were estimated with two models; a crude model only adjusted for the matching factors sex and age, and a multivariate model further adjusted for income-level, educational attainment, centrality of place of residence, and immigration background, as these variables were found to be associated with suicide in Norway (Puzo et al., 2017, 2018; Tang et al., 2019). Incidence density sampling and an uncommon dependent variable makes the ORs approximately equal to the incidence rate ratio (Collett, 1991, pp. 223–276).

Interactions between marital status and sex, age, educational attainment, and income-level were assessed with the log likelihood ratio-test by comparing a model that included all the variables and interaction terms between the variable of interest and each of the other variables to a model where the interaction between the variable of interest and marital status was excluded. In the interaction analyses with income-level and educational attainment, subjects below 25 years were excluded to reduce confounding by ongoing education, and the study period was restricted to 1994–2012, as FD-Trygd database only has income-data from 1993 and onwards. ORs derived from the same analysis were compared with Wald-test using Stata's Test-function. Population attributable risk (PAR) was calculated as described by Bruzzi et al. from the adjusted ORs and the distribution of exposure in the cases (Bruzzi et al., 1985). PAR in this context is the proportion of the total number of suicides in the population that would not have occurred if the suicide rate in the single statuses had been equal to the rate in the married population.

## 3 Results

### 3.1 Distributions

Table 1 shows the distributions of marital status categories among cases and controls. During the 21-year period of study 11 051 persons 18 years and older died by suicide, comprising 8026 (72.6%) males and 3025 (27.4%) females, with a mean age of 45.7 (SD = 18.2) for males and 47.7 (SD = 17.3) for females. A considerably smaller proportion of the cases compared to the controls was married (25.9% vs 47.7%), while larger proportions were in a never married (45.5% vs 38.0%) separated (5.6% vs 1.9%), divorced (15.9% vs 8.5%), or widowed status (7.0% vs 3.1%). Approximately the same proportion of male and female study subjects were married, but relatively more males than females were in a never-married status, while less were in a divorced or widowed status.

**Table 1:** Distribution of marital status categories among cases and controls, and ORs from the conditional regression analyses.

Marital status		Cases (%)	Controls (%)	Crude OR^a^ (95% CI)	Adjusted OR^b^ (95%CI)
**All**					
	Married	2857 (25.9)	88530 (47.7)	1 (ref.)	1 (ref.)
	Never married	5015 (45.4)	70592 (38.0)	3.38 (3.19–3.58)*	2.86 (2.70–3.03)*
	Separated	622 (5.6)	3566 (1.9)	6.37 (5.79–7.00)*	6.06 (5.51–6.68)*
	Divorced	1762 (15.9)	15774 (8.5)	3.66 (3.44–3.90)*	3.35 (3.15–3.58)*
	Widowed	772 (7.0)	5766 (3.1)	2.58 (2.34–2.85)*	2.49 (2.26–2.75)*
**Males**					
	Married	2080 (25.9)	62088 (46.3)	1 (ref.)	1 (ref.)
	Never married	3969 (49.5)	55939 (41.7)	3.42 (3.20–3.65)*	2.67 (2.49–2.86)*
	Separated	443 (5.5)	2487 (1.9)	6.38 (5.70–7.14)*	5.86 (5.23–6.57)*
	Divorced	1102 (13.7)	10209 (7.7)	3.41 (3.15–3.69)*	2.95 (2.73–3.20)*
	Widowed	416 (5.2)	2181 (1.6)	2.80 (2.46–3.19)*	2.64 (2.32–3.01)*
**Females**					
	Married	777 (25.7)	26442 (51.3)	1 (ref.)	1 (ref.)
	Never married	1046 (34.6)	14653 (28.4)	3.23 (2.88–3.62)*	3.36 (2.99–3.77)*
	Separated	179 (5.9)	1079 (2.1)	6.31 (5.29–7.53)*	6.63 (5.55–7.93)*
	Divorced	660 (21.8)	5484 (10.6)	4.17 (3.74–4.66)*	4.38 (3.92–4.90)*
	Widowed	356 (11.8)	3585 (7.0)	2.36 (2.02–2.75)*	2.54 (2.18–2.97)*

Test for interaction between marital status and sex: χ^2^ = 36.34, p < 0.0001. ^a^ Adjusted for the matching factors sex and age. ^b^ Adjusted for the matching factors sex and age, in addition to educational attainment, income-level, centrality of residence, and immigration status.

*p < 0.01.

### 3.2 Suicide risk associated with marital status and differences by sex and age

Results from the crude conditional logistic regression analysis, controlled for sex and age through matching (Table 1), showed that being in a never married (OR 3.38, 95%CI 3.19–3.58), separated (OR 6.37, 95%CI 5.79–7.00), divorced (OR 3.66, 95%CI 3.44–3.90), or widowed (OR 2.58, 95%CI 2.34–2.85) was associated with significantly increased risk of suicide as compared to being married. After further adjustment for educational attainment, income-level, centrality of residence and immigration background these estimates were slightly attenuated for males but augmented for females; more detailed investigation (not showed) showed that inclusion of income and education had the strongest effect. Test of interaction between sex and marital status was significant (p < 0.001), but the differences in ORs between males and females were generally small. The risk associated with being divorced was more pronounced for women than for men, and the risk associated with a status of being separated was substantially higher than the risk for a divorced status for both sexes (p < 0.001), with the difference being most pronounced for males. When looking into the risk by time since separation (Table 2), the results showed that the increased risk weakened with the time passed. The risk was strongest the first 30 days into the separation (OR: 12.76, 95%CI: 8.83–18.46), and slightly less in the next two months (OR: 11.27, 95%CI: 8.57–14.81); it then decreased considerably in the following time periods. After more than a year into the separation suicide risk (OR: 4.65, 95% CI: 4.01–5.19) was only slightly higher than for a divorced status and this difference was only significant for males.

**Table 2:** ORs from the conditional logistic regression analyses for a marital separation.

Days from separation			Adjusted OR (95%CI)^a^			
		All		Males		Females
0-30	12.76 (8.83-18.46)*		13.20 (8.46-20.60)*		12.02 (6.20-23.30)*	
31-92	11.27 (8.57-14.81)*		11.69 (8.55-15.99)*		9.88 (5.61-17.40)*	
93-183	8.54 (6.53-11.17)*		8.08 (5.85-11.17)*		9.72 (5.97-15.81)*	
184-365	7.36 (6.07-8.92)*		7.74 (6.17-9.72)*		6.62 (4.59-9.53)*	
>365	4.56 (4.01-5.19)*		4.21 (3.61-4.91) *		5.59 (4.42-7.07)*	

^a^ Adjusted for the matching factors sex and age, in addition to educational attainment, income-level, centrality of residence, and immigration status

*p < 0.01

**Fig. 1:**
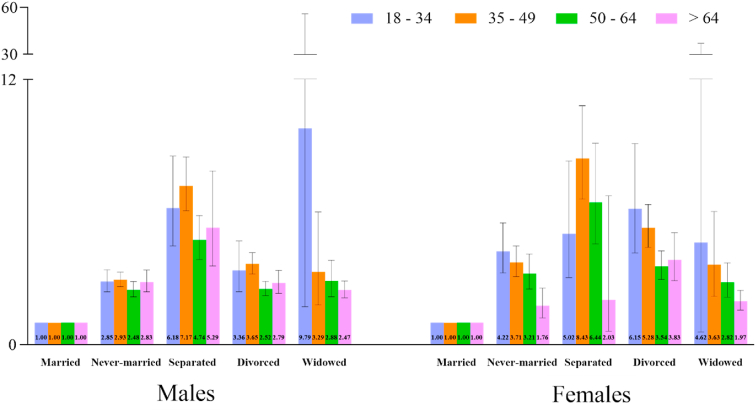
Test of interaction between marital status and age group: Males: χ^2^ = 31.36, p = 0.0049; Females: χ^2^ = 41.42, p = 0.0003

Adjusted ORs stratified by sex and age-group are displayed in Fig. 1. Test of interaction between marital status and age was significant for both males (p = 0.0049) and females (p = 0.0003). For males, there were no large differences between the age-groups, but the overall pattern suggested a higher risk in the lower age-group. For females, the differences were more notable, and the ORs clearly more pronounced in the youngest than in the oldest age-group for all statuses, with the pattern being most salient among the never-married. For both males and females, a separated status seemingly had the strongest effect in the middle-aged.

### 3.2 Interaction with educational attainment and income-level

Table 3 shows the results from the interaction analyses between marital status and income-level and educational attainment stratified by sex. The likelihood ratio-tests showed strong overall interactions between marital status and both covariates for both males and females. The ORs showed that all the single statuses were associated with an increased risk for suicide across all levels of both variables, but some ORs were markedly modified by the interaction terms. Most notably, the increased risk associated with being never married was substantially less increased for males and females with high educational attainment or income. For males, there were no significant differences in income-groups in the other statuses, but females in a divorced status with high income had a relatively smaller increased risk than those with low income (p = 0.007). Among separated, divorced, and widowed, high educational attainment was associated with a more pronounced risk. The differences were small and statistically insignificant for males, while for females, they were more pronounced and the association statistically stronger, although only significant for a widowed status.

**Table 3:** Suicide risk associated with specific marital status by: PANEL A Income and PANEL B Educational attainment. Individuals under the age of 25 were excluded.

**PANEL A**					
**Income**	**< 3 G**^a^	**3–6 G**	**> 6 G**	**Test of interaction**	**Diff low-high**
**Males**					
Married (ref)	2.07 (1.79-2.40)*	1.63 (1.46-1.82)*	ref.	χ2 = 89.07, p < 0.001	
Never married	3.57 (3.11-4.10)*	2.59 (2.35.2.86)*	2.02 (1.76-2.30)*		p < 0.001
Separated	3.90 (2.83-5.37)*	6.80 (5.73-8.05)*	5.65 (4.59-6.95)*		p = 0.056
Divorced	2.96 (2.48-3.54)*	3.32 (2.95-3.72)*	2.50 (2.12-2.93)*		p = 0.165
Widowed	2.56 (2.03-3.24)*	2.83 (2.35-3.40)*	3.44 (2.44-4.85)*		p = 0.165
**Females**					
Married (ref)	2.29 (1.74-3.02)*	1.30 (0.99-1.71)	ref.	χ2 = 55.53, p < 0.001	
Never married	4.43 (3.72-5.28)*	3.39 (2.85-4.03)*	1.64 (1.12-2.40)*		p < 0.001
Separated	7.19 (5.22-9.90)*	6.69 (5.11-8.77)*	5.90 (3.62-9.62)*		p = 0.508
Divorced	5.08 (4.25-6.07)*	4.58 (3.85-5.45)*	2.96 (2.09-4.20)*		p = 0.007
Widowed	2.27 (1.83-2.82)*	3.76 (2.94-4.80)*	3.65 (2.26-5.89)*		p = 0.073

**PANEL B**					
Education	Elementary school	High school	Bachelor and higher		
**Males**					
Married (ref)	1.39 (1.20-1.60)*	1.09 (0.96-1.24)	ref.	χ2 = 95.63, p < 0.001	
Never married	3.05 (2.73-3.42) *	2.75 (2.49-3.05)*	1.91 (1.64-2.24)*		p < 0.001
Separated	5.45 (4.41-6.73)*	6.05 (5.05-7.24)*	6.58 (4.98-8.69)*		p = 0.288
Divorced	2.74 (2.37-3.15)*	3.01 (2.67-3.40)*	3.43 (2.82-4.18)*		p = 0.065
Widowed	2.27 (1.86-2.78)*	3.28 (2.68-4.01)*	3.28–2.22-4.85)*		p = 0.099
**Females**					
Married (ref)	1.05 (0.84-1.31)	0.80 (0.65-0.99)*	ref.	χ2 = 95.60, p < 0.001	
Never married	4.42 (3.63-5.39)*	4.00 (3.32-4.82)*	2.68 (2.14-3.36)*		p < 0.001
Separated	5.58 (4.00-7.79)*	7.96 (5.87-10.80)*	8.58 (5.95-12.36)*		p = 0.088
Divorced	3.84 (3.15-4.69)*	5.40 (4.50-6.47)*	5.24 (4.10-6.70)*		p = 0.054
Widowed	1.99 (1.57-2.52)*	4.06 (3.21-5.14)*	4.90 (3.16-7.59)*		p < 0.001

All odds ratios are controlled for age and sex through matching, in addition to centrality of residence, immigration status and the variables in the table.

^a^ G is a number of Norwegian kroner (NOK) adjusted annually according to the expected wage growth. 1 G was 37 300 NOK in 1993 and 79 216 NOK in 2011

*p<0.01

### 3.3 Population attributable risk (PAR)

Results from the calculations of PAR, shown in Table 4, demonstrated that the increased risk associated with the single statuses contributed to 47.8% of male and 53.3% of female suicide cases during the study period. Notably, PAR for a separated or a divorced status was respectively 4.6% and 9.0% for males, and 5.0% and 16.8% for females. This was chiefly due to the high PAR for these statuses for people in the 35–49 and 50–64 age groups. For males, a separated status contributed 8.5% in the 35–49 group and 5.7% in the 50–64 group, while a divorced status contributed 14.1% and 15.4%. For females, the numbers were even higher: A separated status contributed 10.2% in the 35–49 group and 5.1% in the 50–64 group, while a divorced status contributed 24.3% and 22.6%. Overall, a never-married status contributed most, especially among the youngest (18–34) where the majority have yet to marry. In the oldest group (> 64), PAR was highest for a widowed status, being 14.2% for males and 23.0% for females.

**Table 4:** Population attributable risks (%) for each sex- and age-group, calculated from the adjusted odds ratios and the distribution of cases among the marital status categories.

	18-34	35-49	50-64	≥ 65	All
**Males**					
Never married	58.1	29.9	12.7	10.5	30.9
Separated	1.9	8.5	5.7	2.0	4.6
Divorced	1.4	14.1	15.4	7.9	9.0
Widowed	0.1	0.4	2.2	14.2	3.2
Total	61.4	52.9	36.4	34.7	47.8
**Females**					
Never married	62.0	21.8	8.7	3.0	24.3
Separated	2.4	10.2	5.1	0.3	5.0
Divorced	5.0	24.3	22.6	12.7	16.8
Widowed	0.1	1.4	6.4	23.0	7.1
Total	69.5	57.8	42.8	39.0	53.3

## 4 Discussion

### 4.1 Main findings and interpretations

This register-based population study investigated the relationship between marital status and suicide in the Norwegian population by utilizing interlinked individual-level data from national registers. We found robust evidence of a substantially increased suicide risk associated with all the single statuses, for both genders and in all age-groups, even after adjustment for several relevant socioeconomic factors. Most importantly, we found an alarmingly high risk of suicide for men and women enduring a marital separation, and a status of being separated or divorced accounted for 13.6% male and 21.8% female suicides during the study period. The study also indicated that the protective effect of marriage is strongest for young persons, and that the increased risk is stronger for never-married men and women with low educational attainment or income, for females in a divorced status with low income, and for widows with high educational attainment.

In line with studies from Australia (Cantor & Slater, 1995; Wyder et al., 2009), but contrary to findings from US and Denmark (Agerbo, 2005; Cook, 2019; Kposowa et al., 2020), the risk was substantially higher for persons enduring a marital separation than for people who had undergone the formal divorce, but in our study the increased risk was no less pronounced for females than males. These across-study differences may possibly be accounted for by differences in study design, as all the studies which have not found the risk among the separated to be higher than for the divorced extracted marital status from a time point which preceded the suicide by up to several years. At this point a large proportion of those identified as being in a separated status probably would be divorced or remarried, and conversely, a proportion of those identified as married at baseline would endure a separation during the follow up period. Our finding that suicide risk was especially high in the beginning of the separation period before it leveled off strongly supports the hypothesis that marital dissolution leads to a state of crisis, with the level of psychological stress peaking close to the event. Our finding of a high risk among the divorced, may imply that marital dissolution also could lead to chronic strain, although selection effects both out of marriage and through failing to enter a new marital union may account for some of this risk. The difference in risk between the separated and divorced was larger and statistically stronger for men than women, which parallels findings from studies reporting on adaptation and risk of sickness absence from work after a marital separation (Dahl et al., 2015; Leopold, 2018). This may reflect that women more often initiate separation, which is associated with better adaptation initially, but also run a higher risk of acquiring long-term economic strain. This also fits well with results from the interactions analyses which showed that high income was associated with less pronounced risk for women, but not men. Despite high gender equality in Norway, women more often work part-time (Kjeldstad & Nymoen, 2009), and probably run a larger risk of becoming economically disadvantaged after a marital dissolution. For both males and females, the risk was most pronounced for people in midlife. It is possible that people in this age-group are heavily invested in their relationships, so marital dissolution could entail a relatively large loss of investment and blow to identity (Shiner et al., 2009).

To our knowledge, this study is the first to provide analyses on the interacting effect of educational attainment and income-level on the increased risk associated with each of the single marital statuses. The increased risk was substantially higher for never married men and women with low education or income, but except for a divorced status among women with high income, this buffering effect was not seen for the other statuses. This raises the possibility that the interaction seen for the never married reflects matrimonial selection, rather than a causal effect. When also considering the main effects of the interaction variables, single people with low income stands out as a high-risk group. For instance, a never married status combined with being in the lowest income-group increased suicide risk with 7.39 (95%CI: 6.60–8.28) for males and 10.14 (95%CI: 7.66–13.44) for females compared to being married and in the highest income-group.

Remaining unmarried has gained some popularity in contemporary society, since more people choose to live together in a domestic partnership without being married. The proportion of cohabitants in the Norwegian population increased from 13% in 1993–1995 to 18% in 2011 (SSB, 2015). Cohabitants can attain some of the tax benefits of married couples and gain the same rights and obligations towards each other through a cohabitation agreement. As cohabitation is not a formal status of marriage, cohabitants are lumped into the other statuses in this study. Research from Denmark, a country with many similarities with Norway, has indicated that the suicide risk for cohabitants is slightly higher than for married persons (Qin et al., 2003), but much lower than for those who are single, which may imply that the effect sizes would be higher if cohabitation was controlled for. This means that living without a partner constitutes a considerable risk of completed suicide, and that marriage or cohabitation is a strong protective factor, also in a country and time-period where the religious, cultural, and economic pressures into matrimonial union are reduced, suggesting that the interpersonal aspects of a relationship have important mediating effects.

Stratification by age group showed that the effect of marriage was in general less pronounced with older age, and as cohabitation is much more common among younger persons, the actual trend is probably stronger than the results indicate. Although the differences could be caused by selection effects or cohort differences, these results indicate that some of the social and psychological factors associated with age influences the effect of marriage. Since younger age has been associated with a higher prevalence of depression and anxiety disorders (Hudson, 2012; Jorm, 2000), poorer emotional regulation capacity (Gross et al., 1997; Lawton et al., 1992), and more frequent exposure to stressors from the social environment (Luong et al., 2011), younger persons might be even more in need of the stress reducing effects of social support inherent in most married relationships. The pattern was less pronounced for men than women, which might be because men have a relatively higher risk of impulsive suicide early in life, which could mask age-differences from other causes.

Effect sizes were of approximately the same magnitude for men and women among widowed. This was an unexpected finding, as almost all earlier studies have found the elevated risk to be more pronounced for widowers than for widows. Traditionally, men have gained more instrumental support from their spouse, which make them more vulnerable following marital loss, especially when this happens in old age (Umberson et al., 1992). It is possible that the high gender equality in Norway makes men more prepared for the challenges of living alone compared to widowers in countries with less equality, and also, Norwegian municipalities offer more care and help with house chores for older persons who need it than in many other countries; this could make up for some of the lost instrumental support. However, despite small differences in the relative risk between men and women for all statuses, the difference in baseline rate makes the absolute increase in risk associated with any single status substantially higher for men.

### 4.2 Strengths and limitations

A considerable strength of this study is the use of interlinked individual data from national longitudinal registers with reliable data which covers the entire population and contains insignificant amounts of missing information. This made possible adjustment for several important covariates and eliminates the chance of bias caused by misclassification or by the selection of study subjects. As the registers give access to study subjects marital status at the time of suicide or matching, artificially reduced effect sizes caused by inaccurate information was avoided. In addition, the large data set, and a matched case-control-design produced precise and reliable results and permitted robust subgroup analyses.

On the other hand, the study also has some notable limitations. The nested case-control design adopted in this study efficiently minimized possible effects of time-varying variables that are not considered in the adjustment, but it does not have the capacity to draw conclusions about causality, or to quantify the contribution made by selection effects. Moreover, some important variables that might confound or mediate the effect of singlehood were not included due to the limited availability of data for this study. Most notable are psychiatric disorders, which are strongly linked to both marital status and completed suicide, and parenthood, which protects against suicide and probably mediates some of the effect of marriage, especially for women (Qin & Mortensen, 2003). In the case of marital dissolution, initiator role and child custody are important factors to consider (Amato, 2000). Lastly, the register data we received do not contain information on cohabitation. This may imply an underestimation of the effect sizes and would not have changed our conclusions, but cohabitation has become a common way of living, both pre-marriage and as a substitute for marriage, and hence an important status which should be investigated.

## 4.3 Conclusion and implications

Using data covering the entire national population of Norway, this study provides strong evidence that a status of being single is associated with a substantially increased risk of suicide as compared to a status of being married, and the risk is highest during a marital separation and particularly high right after the separation is filed. Furthermore, the increased risk varies in strength according to sex, age, educational attainment, and income-level.

The study suggests that social support through living in spousal relationships is an important protective factor against suicide. Healthcare workers should be aware the increased suicide risk in people with little social support, particularly if they recently went through a marital dissolution, and should be especially observant those with low income, as they have an increased baseline rate, and those with low education, as they have a higher threshold for utilizing psychiatric health care services (Hansen et al., 2012). Psychological counselling should be offered to people enduring a marital separation soon after the separation is filed.

## Ethical approval

The study was approved by the Regional Committees for Medical and Health Research Ethics and owners of the relevant registers.

## Funding sources

The study received financial support from the Faculty of Medicine at the 10.13039/501100005366University of Oslo and grant was received from the 10.13039/501100005416Norwegian Research Council through the Medical Student Research Program at the University of Oslo. The funding sources was not involved in study design, data collection, analysis and interpretation of data, in the writing of the report, or in the decision to submit the article for publication.

## Author contributions

Erik Oftedahl Næss and Ping Qing conceived the idea of the study. Erik Oftedahl Næss conducted the statistical analyses, interpreted the results, and wrote the original draft. Ping Qin administered the project, prepared the data, and supervised the study. Ping Qin and Lars Mehlum took part in interpretation of results and critically revised the manuscript. All authors read and approved the final version of the paper.

## Declarations of interest

None.

## Data availability statement

This population study was based on individual-level data from The Norwegian Cause-of-Death Register (held by Norwegian Institute of Public Health), Central Population Register (held by the Norwegian Tax Administration) and the Statistics Norway's events database (held by Statistics Norway). The ethical approval of this research project does not include permission to publicly share the raw data. Qualifying researchers can apply for access to relevant data with the Norwegian Institute of Public Health (https://www.fhi.no/en/), the Statistics Norway (https://www.ssb.no/en/omssb/tjenester-og-verktoy/data-til-forskning) and the Norwegian Tax Registry (https://www.skatteetaten.no/en/person/national-registry/) upon the approval from the Regional Committees for Medical and Health Research Ethics (https://helseforskning.etikkom.no/).
